# The Role of Omega-3 Fatty Acids in Acute Pancreatitis: A Meta-Analysis of Randomized Controlled Trials

**DOI:** 10.3390/nu7042261

**Published:** 2015-03-31

**Authors:** Qiu Cheng Lei, Xin Ying Wang, Xian Feng Xia, Hua Zhen Zheng, Jing Cheng Bi, Feng Tian, Ning Li

**Affiliations:** 1 Graduate School, Southern Medical University, Guangzhou 510515, China; E-Mail: lqiuchenggd@163.com; 2 Department of General Surgery, Jinling Hospital, Clinical College of Southern Medical University, Nanjing 210002, China; 3 Research Institute of General Surgery, Jinling Hospital, Medical School of Nanjing University, Nanjing 210002, China; E-Mails: ahbijingcheng@163.com (J.C.B.); easyhard666@163.com (F.T.); liningnju@163.com (N.L.); 4 Department of Surgery, Prince of Wales Hospital, Faculty of Medicine, the Chinese University of Hong Kong, Hong Kong 999077, China; E-Mail: xiaxianf@gmail.com; 5 Key Laboratory for Medical Molecular Diagnostics of Guangdong Province, Guangdong Medical College, Dongguan 523808, China; E-Mail: zhenghzgd@163.com

**Keywords:** acute pancreatitis, omega-3 fatty acids, nutrition support, parenteral nutrition, enteral nutrition, meta-analysis

## Abstract

To determine whether treatment with omega-3 fatty acids (ω-3 FA) provides benefits to patients with acute pancreatitis (AP). The Cochrane Library, PubMed, Embase, Web of Science, and Chinese Biomedical Literature Database were searched. Data analysis was performed using Revman 5.2 software. A total of eight randomized controlled trials (RCTs) were included. Overall, ω-3 FA treatment resulted in a significantly reduced risk of mortality (RR 0.35; 95% CI 0.16 to 0.75, *p* < 0.05), infectious complications (RR 0.54; 95% CI 0.34 to 0.85, *p* < 0.05) and length of hospital stay (MD –6.50; 95% CI −9.54 to −3.46, *p* < 0.05), but not length of ICU stay (MD −1.98; 95% CI −6.92 to 2.96, *p* > 0.05). In subgroup analysis, only patients who received *ω*-3 FA parenterally had some statistically significant benefits in terms of mortality (risk ratio (RR) 0.37; 95% confidence interval (CI) 0.16 to 0.86, *p* < 0.05), infectious complications (RR 0.5; 95% CI 0.28 to 0.9, *p* < 0.05) and length of hospital stay (mean difference (MD) −8.13; 95% CI −10.39 to −5.87, *p <* 0.001). The administration of ω-3 FA may be beneficial for decreasing mortality, infectious complications, and length of hospital stay in AP, especially when used parenterally. Large and rigorously designed RCTs are required to elucidate the efficacy of parenteral or enteral ω-3 FA treatment in AP.

## 1. Introduction

Acute pancreatitis (AP) is a common immuno-inflammatory disorder of the pancreas. Nearly 20% of AP patients evolve into severe acute pancreatitis (SAP), and, simultaneously, the mortality rate increases to 20% [1,2,3]. Excessive acinar cell injury and amplified inflammatory responses can lead to systemic inflammatory response syndrome (SIRS) [4] and infection of pancreatic necrosis, giving rise to distant organ damage and multiple organ dysfunction syndrome (MODS), which are the main causes of death from AP [5,6]. Hence, attempts to enhance immune function, suppress the hyper inflammatory responses and reestablish tissue and organ homeostasis in AP patients have been made in clinical practice [7].

Accumulating evidence has suggested that omega-3 fatty acids (ω-3 FA) can alter cytokine production, modulate inflammatory and immunological response [8,9] and thus be expected to lower the rates of infectious complications, shorten the hospital stay in the intensive care unit (ICU) or on regular medical wards as well [10]. Anti-inflammatory and immunomodulatory effects of ω-3 FA may provide an important therapeutic option for the patients with AP. Nonetheless, according to the current evidence, the role of ω-3 FA treatment in AP patients remains unclear [11].

Some studies demonstrated that nutritional support in patients with AP, either from the parenteral or enteral route, could significantly reduce mortality [12]. However, results from randomized controlled trials (RCTs) assessing the effects of ω-3 FA administrated by parenteral nutrition (PN) or enteral nutrition (EN) in AP patients were inconsistent. In 2008, a meta-analysis reported that EN supplemented with ω-3 FA had no beneficial effects on infectious complications, mortality and length of hospital stay in AP patients [13]. But the sample size of this meta-analysis was fairly small and the analysis excluded studies of PN. Another meta-analysis concluded that immunonutrients like ω-3 FA added to PN can improve the prognosis in patients with AP [14]. Recently, an increasing number of RCTs about ω-3 FA treatment in AP patients have been performed. However, most of these have been insufficiently powered to detect significant results. We therefore decided to systematically review the clinically meaningful outcomes of RCTs that investigated the role of omega-3 fatty treatment for patients with AP.

## 2. Methods

### 2.1. Literature Search

The Cochrane Library, PubMed, Embase, Web of Science, and Chinese Biomedical Literature Database were searched to identify RCTs of interest from inception to October 2014. We used key words including “omega-3 fatty acids”, “fish oil,” “polyunsaturated fatty acids”, “eicosapentaenoic acids”, “docosahexaenoic acids” “DHA”, “EPA”, “PUFA” paired with the following: “acute pancreatitis”, “severe pancreatitis”, “acute hemorrhagic pancreatitis”or “acute necrotizing pancreatitis”, “severe acute pancreatitis”. No language restrictions were placed on the searches. Abstracts had been accepted for inclusion into this review if the data was available.

### 2.2. Study Selection

All RCTs evaluating the effects of the ω-3 FA administration on AP were included, regardless of the strategy of nutrition support. Review articles were used to identify additional relevant studies. Individual authors were contacted via e-mail when additional information about their studies was required for the purpose of our meta-analysis. If the information was still unavailable due to no response from the author or data loss, we reported the available results as stated in the trial report. The outcomes observed were mortality, infectious complications, the length of hospital stay and the length of ICU stay. We used 28-day mortality if the hospital mortality was not reported. The studies that did not report at least one of the outcomes of interest were excluded. Two authors independently performed the literature search, evaluation of trials, quality assessment and data extraction according to the inclusion criteria. Discrepancies were resolved by consensus.

### 2.3. Quality Assessment

Qualities of included studies were judged by two independent researchers according to the Cochrane risk of bias guidelines [15], which included random sequence generation, allocation concealment, blinding of participants, personnel and outcome assessors, incomplete outcome data, selective outcome reporting, and other bias.

### 2.4. Statistical Analysis

The data analysis was performed using Revman 5.2 software (Cochrane IMS, Oxford, UK). The outcomes for categorical variables were aggregated to obtain a pooled risk ratio (RR) with a 95% confidence interval (CI). For continuous variables, the pooled effect was reported as a weighted mean difference (MD) with the corresponding 95% CI. Heterogeneity was assessed using *I^2^* and χ^2^ tests, and a *p* value < 0.10 considered to be significant. If the heterogeneity was present, the pooled effect size was calculated through a random-effects model; otherwise, a fixed-effects model was applied. Forest plots were constructed with *p* < 0.05 considered to be significant. A subgroup analysis was performed according to the strategy of nutrition support (parenteral *vs.* enteral). A sensitivity analysis was used to assess the sources of heterogeneity.

## 3. Results

### 3.1. Search Results and Study Characteristics

**Figure 1:**
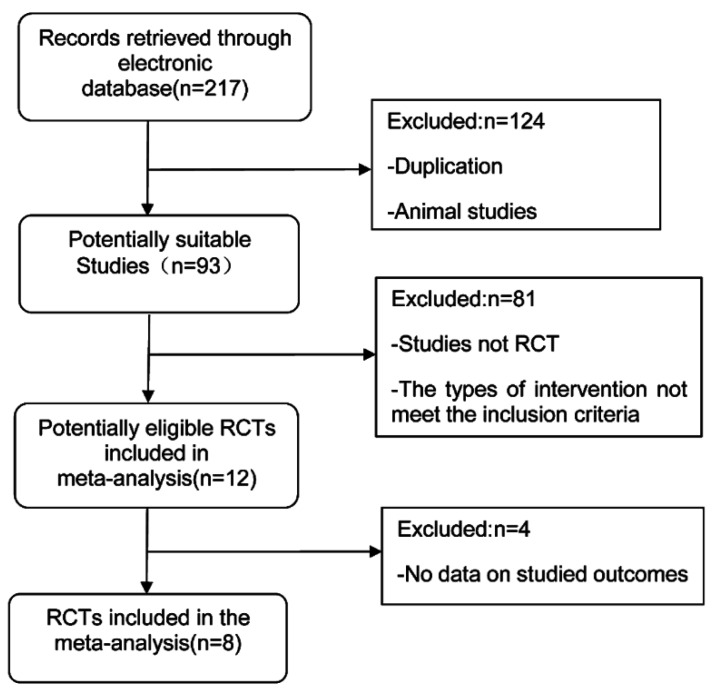
Flow chart illustrating the study selection process.

**Figure 2:**
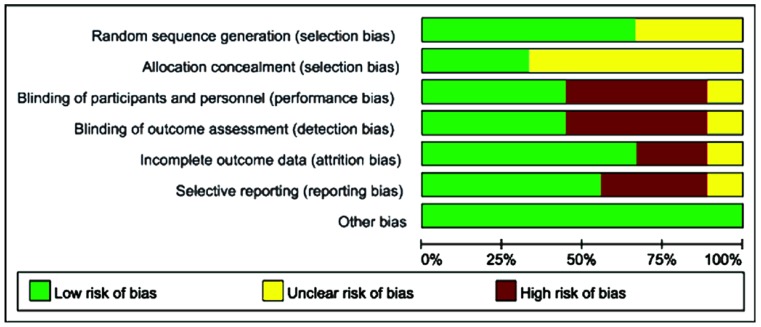
Methodological quality of RCT included in this meta-analysis.

The database search yielded 217 records that were subsequently screened. Review articles, viewpoints, editorials, commentaries, and animal studies were excluded. Studies that did not provide data on the effect of ω-3 FA on AP were also excluded. Ultimately, eight RCTs [16,17,18,19,20,21,22,23] fulfilling the criteria for consideration in our meta-analysis (Figure 1), with a total of 364 subjects were included. The average sample size of each study were 45 subjects (range: 28–64 subjects). One hundred and eighty-one patients were randomized to ω-3 FA treatment and 183 patients to the control group. Seven studies administered PN while two studies used EN. One study administered ω-3 FA enterally in combination with other nutrients (glutamine and arginine). The ω-3 FA dose varied from 0.15 g (kg day)^−1^ to 0.2 g (kg day)^−1^ in four studies and the dosage of EPA + DHA was 2.84g day^−1^ in one study. A summary of study characteristics is presented in Table 1. The risk of bias assessments showed that most of these studies were of moderate quality (Figure 2).

### 3.2. Effect of ω-3 FA on Mortality

**Figure 3:**
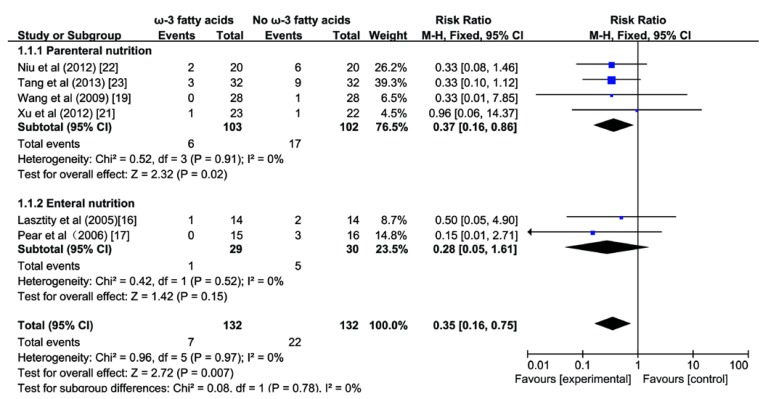
Forest plot of the effect of omega-3 fatty acids on mortality in acute pancreatitis. CI, confidence interval; df, degrees of freedom; MH, Mantel-Haenszel (statistical method).

A pooled analysis of 6 studies [16,17,19,21,22,23] enrolling 264 patients with AP revealed that the administration of ω-3 FA significantly reduced mortality (RR 0.35; 95% CI 0.16 to 0.75, *p <* 0.05; Figure 3). There was no heterogeneity among the studies (*I^2^* = 0%, *p* = 0.97). In the subgroup analysis, the beneficial effect of ω-3 FA on mortality was statistically significant in patients receiving PN (RR 0.37; 95% CI 0.16 to 0.86; *p <* 0.05; Figure 3), while those who received EN had no obvious benefit (RR 0.28; 95% CI 0.05 to 1.61; *p* > 0.05). There was no difference between two subgroups (*I^2^* = 0%, *p* = 0.78).

**Table 1:** Study characteristics for the included studies.

Study	Country	Year	Type	No. of Patients	Patients (*ω*-3/con)	Mean age (*ω*-3/con)	Male/ Female	Severity Criteria of Used	Intervention	Route of Nutrition	Dose	Duration of Intervention
Lasztity *et al.* [16]	Hungary	2005	RCTFull text	28	14/14	56.1/55.9	16/12	APACHE II score ≥ 5	ω-3 FA alone	EN	2.84 g day^−1^^a^	5–7 days
Pearce *et al.* [17]	UK	2006	RCT DBFull text	31	15/16	63.2/73.2	18/13	APACHE II score ≥ 8	ω-3 FA composite	EN	NR	3–15 days
Wang *et al.* [18]	China	2008	RCTFull text	40	20/20	37/40	28/12	Mean APACHE II score = 12.5	ω-3 FA alone	PN	0.15–0.2g (kg day)^−1^^b^	5 days
Wang *et al.* [19]	China	2009	RCTFull text	56	28/28	40/42	39/17	Mean APACHE II score = 14	ω-3 FA alone	PN	0.15–0.2g (kg day)^−1^^b^	5 days
Xiong *et al.* [20]	China	2009	RCTFull text	60	30/30	41.2/42.7	36/24	APACHE-II ≥ 8; Ranson's score ≥ 3; Balthazar CT severity score > 6	ω-3 FA alone	PN	0.2 g (kg day)^−1^^b^	2 weeks
Xu *et al.* [21]	China	2012	RCTFull text	45	22/23	50.5/51.7	30/15	APACHE II score > 8; Ranson score > 3	ω-3 FA alone	PN	0.2 g (kg day)^−1 b^	2 weeks
Niu *et al.* [22]	China	2012	RCTFull text	40	20/20	Mean 50	25/15	APACHE II score ≥ 8	ω-3 FA alone	PN	NR	5 days
Tang *et al.* [23]	China	2013	RCTFull text	64	32/32	Mean 37.5	50/24	APACHE II score ≥ 8; Ranson’s score ≥ 3	ω-3 FA alone	PN	NR	5 days

ω-3—omega-3 fatty acids (ω-3 FA) group; con—control group; RCT—randomized controlled trial; DB—double blind; NR—not reported; APACHE—Acute Physiology and Chronic Health Evaluation; EN—enteral nutrition; PN—parenteral nutrition; a—the dosage of EPA + DHA; b—the dosage of ω-3 omega-3 fatty acids.

### 3.3. Effect of ω-3 FA on Infectious Complications

**Figure 4:**
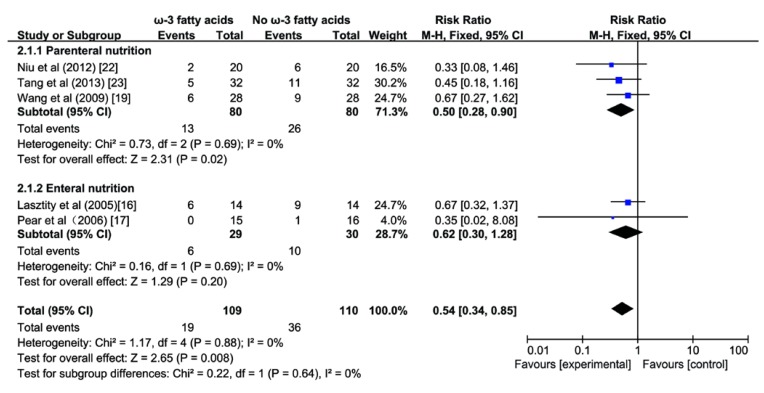
Forest plot of pooled estimates of omega-3 fatty acid supplementation on infectious complications in acute pancreatitis. CI, confidence interval; df, degrees of freedom; MH, Mantel-Haenszel (statistical method).

A pooled analysis of five studies [16,17,19,22,23] enrolling 219 patients with AP revealed a significant reduction of infectious complications was observed in the ω-3 FA group compared with the control group (RR 0.54; 95% CI 0.34 to 0.85; *p <* 0.05; Figure 4), and no significant heterogeneity was found among the studies (*I^2^* = 0%, *p* = 0.88). A subgroup analysis indicated a significant reduction of infectious complications in patients receiving PN (RR 0.50; 95% CI 0.28 to 0.9; *p <* 0.05; Figure 4) compared to those fed enterally (RR 0.62; 95% CI 0.3 to 1.28; *p* > 0.05; Figure 4). There was no difference between the two subgroups (*I^2^* = 0%, *p* = 0.64).

### 3.4. Effect of ω-3 FA on Length of Hospital Stay

**Figure 5:**
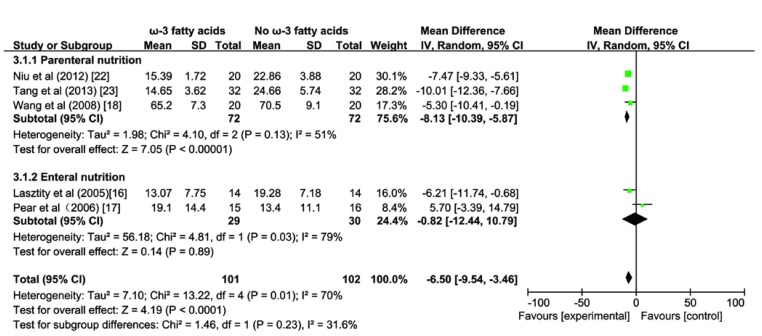
Forest plot of pooled estimates of omega-3 fatty acid supplementation on length of hospital stay in acute pancreatitis. CI, confidence interval; df, degrees of freedom; IV, inverse variance (statistical method).

A pooled analysis of five studies [16,17,18,22,23] enrolling 203 patients with AP revealed that the length of the hospital stay was significantly reduced in the ω-3 FA group compared with the control group (MD −6.50; 95% CI −9.54 to −3.46, *p <* 0.05; Figure 5), and the test for heterogeneity was significant (*I^2^* = 70%; *p* = 0.01). In the subgroup analysis, beneficial effects of ω-3 FA on the reduction of the length of hospital stay were statistically significant for the PN subgroup (MD −8.13; 95% CI −10.39 to −5.87, *p <* 0.05; Figure 5); however, no difference was observed in EN subgroup (MD −0.82; 95% CI −12.44 to 10.79, *p* > 0.05; Figure 5). The test for subgroup differences were not significant (*I^2^* = 31.6%, *p* = 0.23).

### 3.5. Effect of ω-3 FA on Length of ICU Stay

**Figure 6:**
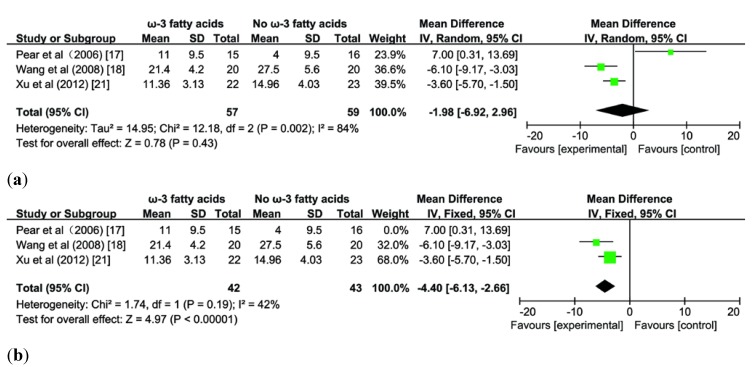
(**a**) Forest plot of pooled estimates of omega-3 fatty acid supplementation on length of ICU stay in acute pancreatitis. CI, confidence interval; df, degrees of freedom; IV, inverse variance (statistical method); (**b**) Forest plot of pooled estimates of omega-3 fatty acid supplementation on length of ICU stay in acute pancreatitis. CI, confidence interval; df, degrees of freedom; IV, Inverse Variance (statistical method).

A pooled analysis of three studies [17,18,21] enrolling 116 patients with AP revealed ω-3 FA had no effect on reducing the length of ICU stay (MD −1.98; 95% CI −6.92 to 2.96, *p* > 0.05; Figure 6a), and there was significant heterogeneity across all studies (*I^2^* = 84%, *p* = 0.002). However, when a sensitivity analysis was done excluding the studies of EN-fed patients [17], the effect of ω-3 FA administrated parenterally on reducing ICU stays was detected (MD, −4.40; 95% CI −6.13 to −2.66, *p <* 0.05; Figure 6b) and there was no statistically significant heterogeneity (*I^2^* = 42%, *p* = 0.19).

## 4. Discussion

According to the present meta-analysis, ω-3 FA has beneficial effects on mortality, infectious complications and length of hospital stay in patients with AP. Moreover, subgroup analysis and sensitivity analysis demonstrated that PN administration of omega-3 acids resulted in a statistically significant reduction of mortality, infectious complications, length of hospital stay and ICU stay in those patients.

Although several meta-analyses have been conducted recently in an effort to clarify whether the administration of ω-3 FA improves outcomes in patients with AP, definitive conclusions have been lacking. Therefore, perspectives on the use of ω-3 FA as treatment in critically ill patients remained conflicting. A meta-analysis evaluating the parenteral use of fish oil lipid emulsions in critically ill patients concluded that fish oil was associated with beneficial effects on clinical outcomes [24]. Conversely, a systematic review indicated that the parenteral supplementation of ω-3 FA did not improve mortality, infectious complications, or the length of ICU stay in critically ill adults, in comparison with standard PN [25]. Controversial results were also reported in a double-blind, multicenter trial conducted RCT performed by Rice *et al.* [26] and Petrov *et al.* [13] that evaluated the effects of enteral immunonutrition (including ω-3 FA) in patients with AP or an acute lung injury. Because the present meta-analysis focused on the effects of ω-3 FA on the clinical outcomes of patients with AP, specifically, the results may be more valid for this patient population.

ω-3 FA suppresses the inflammation and improves the course of infection by reducing proinflammatory eicosanoid and cytokine production [26,27] The ability of ω-3 FA to regulate these immune processes has been well described in many experimental and clinical studies [28,29,30,31,32,33]. Thus, the beneficial clinical outcomes resulting from the administration of ω-3 FA in our meta-analysis may be related to their modulation of inflammation in AP patients.

Our subgroup analysis revealed a statistically significant benefit from ω-3 FA in patients receiving PN instead of those feeding from the enteral route. One reason for this difference may be the small number of RCTs for the EN subgroup; only two RCTs evaluated EN with a total sample size of only 59. This difference may also be associated with the use of EN itself for the administration of ω-3 FA. An RCT that included 272 patients with an acute lung injury found that enteral supplementation of ω-3 FA did not improve systemic inflammation or clinical outcomes [26]. Furthermore, the benefits of EN in critical care patients have been questioned. An RCT in critically ill patients [34] demonstrated impaired gut permeability on day 3 in the EN group. Likewise, another RCT [35] in patients with predicted SAP showed significantly increased intestinal permeability by day 4 in the EN group compared with the PN group. Because immunonutrition with ω-3 FA usually requires a gradual increase in the infusion rate during the first 2–3 days in order to reach the target rate, the amount of omega-3 fatty acid substrate administered through the EN route in the first couple days may be inadequate to produce a prompt modulation of the host response [13]. Thus, the short-term beneficial clinical effects of ω-3 FA may not be significant.

In addition, the sensitivity analysis that was performed after excluding one study [17] of EN-fed patients showed a positive effect on the length of ICU stay in the PN group. It is possible that omega-3 fatty acid administration has better bioavailability with the parenteral route than with the enteral route [24]. Moreover, Pearce *et al*. [17] also reported that the length of hospital stay was considerably longer with EN supplementation of ω-3 FA (11 days *vs.* 4 days in the PN group); however, because of the small sample size, the difference in hospital stay was not statistically significant. This may be related to the nutrient composition of EN, which included the three ingredients of glutamine, arginine, and ω-3 FA; data on the proportion of ω-3 FA were not available. Several epidemiological observations and clinical trials have shown that PN-administered ω-3 FA can modulate the production of proinflammatory cytokines, ameliorate the course of infections, and restore liver function [8,36,37,38]. With regard to the cascade of inflammatory responses during the acute phase of SAP, PN with ω-3 FA may be an optional treatment for patients with AP complicated by multiple organ failure [39]. Wang *et al*. [18] conducted an RCT that assessed the role of PN plus ω-3 FA in SAP patients and demonstrated that PN plus ω-3 FA improved respiratory function and shortened the continuous renal replacement therapy time compared with standard therapy. These results suggest that the systemic response to pancreatic and organ injury is attenuated by omega-3 fatty acid supplementation. Although EN is the preferred route for nutrition delivery, the collective results from previous studies are similar to the present findings regarding the beneficial effects of ω-3 FA supplementation by PN in patients with AP. Future high-quality, large RCTs that compare ω-3 FA administration by PN *vs.* EN in patients with AP are warranted.

The present review has certain limitations. First, the methodological quality of the included RCTs was only moderate. Because our study included some trials with incomplete data, potential bias may distort the findings. Second, we did not analyze the formula composition for patients receiving EN or PN, as these data were not available. Third, the clinical data on SAP were not homogenous; for example, the Acute Physiology And Chronic Health Evaluation II score of the enrolled patients was ≥5 in one study and >8 in another study. Lastly, the sample size in our meta-analysis was small; only 3–6 RCTs were included for each outcome. Our results should be interpreted with caution, because of the lack of study power. While the observations of our meta-analysis are promising, larger RCTs are required to confirm our findings.

## 5. Conclusions

In conclusion, this meta-analysis indicated that patients with AP who received ω-3 FA supplementation may experience some clinical benefits, including reductions in the risk of mortality, infectious complications, and hospital stay. Moreover, the parenteral route of ω-3 FA supplementation may be more beneficial than the enteral route. However, additional large-scale, rigorously designed, multicenter, prospective RCTs are necessary to prove these findings.
